# Heroic Helping: The Effects of Priming Superhero Images on Prosociality

**DOI:** 10.3389/fpsyg.2018.02243

**Published:** 2018-11-23

**Authors:** Daryl R. Van Tongeren, Rachel Hibbard, Megan Edwards, Evan Johnson, Kirstin Diepholz, Hanna Newbound, Andrew Shay, Russell Houpt, Athena Cairo, Jeffrey D. Green

**Affiliations:** ^1^Department of Psychology, Hope College, Holland, MI, United States; ^2^Department of Psychology, Virginia Commonwealth University, Richmond, VA, United States

**Keywords:** heroes, meaning in life, prosocial, helping, priming

## Abstract

Two experiments examined how exposure to superhero images influences both prosociality and meaning in life. In Experiment 1 (*N* = 246) exposed individuals to scenes with superhero images or neutral images. Individuals primed with superhero images reported greater helping intentions relative to the control group, which, in turn, were associated with increased meaning in life (indirect effect only; no direct effect). In Experiment 2 (*N* = 123), individuals exposed to a superhero poster helped an experimenter in a tedious task more than those exposed to a bicycle poster, though no differences were found for meaning in life. These results suggest that subtle activation of superhero stimuli increases prosocial intentions and behavior.

## Introduction

“Unconsciously we all have a standard by which we measure other men, and if we examine closely we find that this standard is a very simple one, and is this: we admire them, we envy them, for great qualities we ourselves lack. Hero worship consists in just that. Our heroes are men who do things which we recognize, with regret, and sometimes with a secret shame, that we cannot do. We find not much in ourselves to admire, we are always privately wanting to be like somebody else. If everybody was satisfied with himself, there would be no heroes.”

—Mark Twain

Heroes play important roles at the intrapersonal, interpersonal, and cultural levels. For many, heroes are inspirational moral exemplars that demonstrate extraordinary courage and live profoundly meaningful lives. Since the earliest literary works, heroes have been extolled and worshipped (e.g., Epic of Gilgamesh), and they occupy a valuable place in many societies. Heroes typically are paragons of prosociality, often going to great lengths to help others, even when doing so endangers themselves or comes at a high cost. But do their prosocial examples influence prosocial actions, and if so, how? In two experiments, we explored whether the subtle activation of heroic images increases prosocial intentions and behaviors, and whether those prosocial inclinations helped enhance one’s perception of meaning in life.

### A Function of Heroes

Heroes serve a variety of social and cultural functions. Previous research has highlighted how heroes may serve an important motivational role of inspiring individuals toward prosocial or altruistic actions (Franco et al., 2016). One of the functions of heroes is to model certain moral behaviors that others should emulate (Kinsella et al., 2015a). Heroes demonstrate behaviors that align with moral principles and inspire individuals to live meaningful and purposeful lives (Allison and Goethals, 2011). Indeed, researchers have converged on the idea that heroism may play an important role in the pursuit and experience of a meaningful life (Green et al., 2017; Kinsella et al., 2017). Thus, heroes are often extolled as morally virtuous individuals who live, or have lived, meaningful lives.

This research suggests that heroes may affect individuals’ perceptions on two related domains. First, because heroes are moral exemplars (Kinsella et al., 2015a,b), exposure to heroes may affect people’s intentions to act prosocially (Franco et al., 2016). Because heroes are defined, in part, by their moral courage and conviction, and their ability to do the right thing in the face of considerable adversity, we suspect that heroes play an important role in prosocial processes. Second, heroes live meaningful lives (Green et al., 2017; Kinsella et al., 2017) and inspire others to do the same (Allison and Goethals, 2011). Some have argued that the motivation for meaning leads to affirmation of heroes, contending that there is a strong link between perceptions of meaning and heroes (Coughlan et al., 2017). Prior work has linked prosociality and meaning in life (Van Tongeren et al., 2016; Klein, 2017), as well as documented that specific prosocial or virtuous behaviors, such as helping others or expressing gratitude, provide a sense of meaning (e.g., Van Tongeren et al., 2015). Accordingly, it is possible that the prosocial nature of heroes is precisely what makes their lives so meaningful. Their sacrifice and prioritization of the needs of others gives their existence meaning. Thus, we sought to focus on how heroes affect prosociality and perceptions of meaning in life.

### The Motivational Function of Heroes

Heroes can be either actual individuals who lived meaningful lives or abstract archetypes that are embedded in myths, folklore, or comic books. Both types of heroes likely possess similar characteristics of virtue and valor. Fictional superheroes, such as Superman, embody courage and conviction, and they are moral exemplars who help, defend, or save those in need (Kinsella et al., 2015b). However, their lofty, otherworldly example is often unattainable. According to social comparison theory (Festinger, 1954), when making an upward comparison toward those that outperform people on a particular domain (e.g., morality), people are motivated to emulate such behaviors when there is little psychological closeness (or in this case, realism), but they may feel threatened when there is greater psychological closeness (or realism). Put another way, we hypothesize that the realism or psychological closeness of the hero will determine whether an assimilation versus contrast effect occurs (Suls et al., 2002). Superheroes may, then, represent an ideal (prosocial) motivational standard for individuals. That is, when being reminded of a superhero, individuals can protect their self-esteem and preserve self-evaluation by recalling that such individuals are not real; thus, they pose little psychological threat and are instead inspirational, motivating them to emulate their noble actions.

Looked at differently, heroes represent part of an individual’s ideal self, at least in some domains (Sullivan and Venter, 2005, 2010). When the representation of this is abstract and embodied in a fictional superhero, individuals may be motivated toward achieving this ideal self. Accordingly, abstract heroes should be particularly motivational (Tesser, 1988) and should prompt individuals to aspire toward their characteristics. We focused on how a reminder of superheroes may elicit greater prosocial intentions and behaviors, which, in turn, may provide individuals with a sense of meaning in life. Given that superheroes are ubiquitous in societies today (e.g., comic book superhero movies have earned billions per year recently, and related television shows have proliferated), this investigation is timely and pertinent.

## Overview and Hypothesis

Based on previous research, superheroes embody aspirational lives that are fictional and unattainable; accordingly, superheroes likely serve a motivational feature as they are exemplars of prosocial and meaningful lives. Our central prediction was that priming abstract superhero images should result in increased prosocial behavior, which, in turn, should be associated with greater meaning in life. Specifically:

*Hypothesis 1:* Exposure to superhero images will increase prosocial behavior.
*Hypothesis 2:* Exposure to superhero images will increase meaning in life.
*Hypothesis 3:* The effect of exposure to superhero images on meaning in life will occur via increased prosocial behavior (i.e., increased prosocial behavior will mediate the influence of exposure to superhero images on meaning in life).

Previous research has identified numerous methodological challenges with studying heroes, and researchers have suggested that experimental methodology is necessary to advance research in this domain (Franco et al., 2016). Toward that end, we designed two experiments to test our central hypothesis. In Experiment 1, participants were exposed to superhero-related versus neutral images, then completed assessments of behavioral intentions to help and reported their meaning in life. In Experiment 2, participants completed tasks in a laboratory room that had a picture of a superhero versus a neutral image, and they subsequently were asked to help the experimenters with a task, then completed an assessment of meaning in life.

In all experiments, we report all conditions and all measures, as well as whether or not any participants were excluded from the analysis. For all studies, we sought to obtain enough participants to detect a medium effect with an alpha of 0.05 (Cohen, 1992). This translated to 64 participants per condition (*N* = 128) for Experiment 1, so we gathered more than 200 participants. In Experiment 2, 87 participants were required; thus, we sought at least 120 participants and continued collecting data until the conclusion of the academic semester. The studies were carried out in accordance with the recommendations of the Belmont Report and the Collaborative Institutional Training Initiative guidelines. The protocol was approved by the Human Subjects Review Board at Hope College. All subjects gave electronic (Study 1) or written (Study 2) informed consent prior to participation.

## Experiment 1: Prosocial Intentions

### Method

Participants were 246 community members (110 females, 136 males) recruited from Amazon’s Mechanical Turk who completed the study for financial compensation. Data from four participants were excluded because they skipped the priming induction. All procedures were completed online. Participants read and agreed to a consent form informing them that their participation was voluntary and that they could quit at any time without penalty. We randomly assigned participants to the superhero condition (*n* = 123) or neutral condition (*n* = 119). In both conditions, participants viewed four everyday household scenes: two desks, one garage and one bedroom. Participants were instructed to find four specific objects in each picture and write one word describing each object once they found it. They were instructed to spend no more than 30 s on each scene. Though each scene contained enough objects to make the task engaging, each object could be easily found in the 30 s timeframe. In the superhero prime condition, the pictures were edited so that one of the target objects in each scene contained an easily recognizable superhero logo or image (e.g., Superman, Spiderman). This acted as a subtle superheroes prime. The scenes otherwise were identical to the control condition (which did not have any superhero images).

Participants next completed a self-reported altruism scale (SRAS; Rushton et al., 1981; α = 0.90), and a self-reported virtues scale (Berry et al., 2005; α = 0.93). We assessed helping intentions whereby participants read six scenarios and indicated their likelihood to help on a 100-point scale (0 = *definitely would not help* to 100 = *definitely would help*). The scenarios involved helping a stranded motorist, recovering a lost dog, donating to charity, returning lost money, shoveling an elderly neighbor’s snowy driveway, and helping a lost stranger with directions. The six items were averaged for composite helping intentions score (α = 0.74).

Meaning in life was assessed using the widely used Meaning in Life Questionnaire (Steger et al., 2006), which is a 10-item self-report measure assessing the presence of meaning (5 items; α = 0.93) and search for meaning (α = 0.96). Consistent with our primary hypothesis, we focused on the presence of meaning.

Finally, we assessed participants’ knowledge of and interest in superheroes via three items: “How would you rate your knowledge of popular superheroes?” (assessed on a 4-point scale), “How much time do you spend watching/reading/playing superhero-related entertainment?” (assessed on a 7-point scale), and “How interested are you generally in superheroes?” (assessed on a 7-point scale). These items were averaged to calculate a mean interest in superheroes (*a* = 0.76; *M* = 3.04, *SD* = 1.11). Participants were also asked to name three of the superheroes used in the study. Nearly every participant named all three superheroes correctly, and every participant correctly identified at least one of the three. Finally, participants indicated what they thought the nature of the study was about (no participants guessed correctly) and were fully debriefed.

### Results

First, to test Hypothesis 1, we examined the effect of the superhero priming condition on helping intentions. To ensure that our results were not simply an artifact of how familiar participants were with the priming stimuli or generally interested in superheroes, we statistically controlled for prior knowledge of superhero characters. In support of Hypothesis 1, participants primed with the superhero images (*M* = 65.27, *SE* = 1.69) reported significantly higher helping intentions than those primed with the neutral images (*M* = 60.45, *SE* = 1.69), *F*(1,236) = 4.07, *p* = 0.045, partial eta^2^ = 0.02. (This effect was marginal when not controlling for superhero knowledge, [*F*(1, 237) = 3.57, *p* = 0.060, partial eta^2^ = 0.02].

Hypothesis 2 was not supported: there were no differences on meaning in life between participants primed with the superhero images (*M* = 4.80, *SE* = 0.15) and those primed with the neutral images (*M* = 4.93, *SE* = 1.14), *F*(1,236) = 0.44, *p* = 0.508, partial eta^2^ = 0.00. However, we proceeded to test the mediation hypothesis (Hypothesis 3) because it is not uncommon to find a significant indirect effect (in this case, from superhero prime to meaning via helping intentions) even in the absence of a direct effect, and testing these indirect effects is vital to theory development (Hayes, 2009).

Data were analyzed using PROCESS (Hayes, 2012) to test an indirect effects model (i.e., Hypothesis 3) from the priming condition to meaning in life via helping intentions across 5,000 bootstrapping iterations. Critically, there was a significant indirect effect from priming condition to meaning in life through helping intentions (completely standardized estimate = 0.03, *SE* = 0.02, 95% *CI* = 0.001 to 0.078), in support of Hypothesis 3. This suggests that priming individuals with abstract superhero images is associated with increased behavioral intentions to help, which, in turn, is associated with greater meaning in life.

We also examined whether the effect on helping intentions was moderated by trait level differences in prosociality. The effect on condition on helping intentions was not moderated by self-reported virtuousness [*F*(1,223) = 0.25, *p* = 0.615] or self-reported altruism [*F*(1,224) = 0.69, *p* = 0.408]. Thus, the prime appears to similarly influence people of varying levels of dispositional prosociality. Although, the prime did not directly affect self-reported virtuousness [*F*(1,227) = 1.29, *p* = 0.258], those in the superhero prime condition self-reported greater altruism (*M* = 3.74, *SD* = 0.66) than those in the neutral condition (*M* = 3.55, *SD* = 0.76), *F*(1,225) = 4.05, *p* = 0.045, which is consistent with the findings regarding self-reported helping intentions.

### Discussion

Even relatively superficial exposure to symbols of heroes increases perceptions of helpfulness and meaning in life. The results of Experiment 1 demonstrated that implicit priming of superhero images was associated with increased self-reported helping intentions. It also revealed a significant positive correlation between helping intentions and meaning in life, suggesting that those participants that reported higher helping intentions also reported significantly higher meaning in life. These results confirm our hypothesis that an abstract superhero prime would increase prosocial behavior in participants and therefore increase their meaning in life. The priming of heroic symbols had an indirect effect on meaning in life through helping intention (though the direct effect from heroic symbols to meaning in life was not significant). Heroic symbols appear to enhance participants’ reporting of greater helping intentions and meaning in life.

One possible drawback of Experiment 1 was its reliance on self-reported helping intentions. We sought to address this limitation in Experiment 2 by examining actual helping behavior. Experiment 2 also used a different (and arguably more ecologically valid) prime of heroic symbols.

## Experiment 2: Prosocial Behavior

### Method

Participants were 123 students (84 females, 36 males, 3 did not report) enrolled in introductory psychology courses at a small, private Midwestern college. No data were excluded. After providing consent, participants were ushered into a small laboratory room with a small poster of Superman (superhero priming condition; *n* = 62) or a bicycle (neutral condition; *n* = 61) affixed to the wall, with a note indicating that this poster was for a different “media images” study, run by a different faculty member in the department, and was to be left undisturbed. Superman was selected because he is a very well-known superhero in American culture. The neutral condition room included a poster of a bicycle–which was similar in color and size to the Superman poster–affixed to the wall.

Participants completed a paper packet with the materials used in Experiment 1, with a notable addition. To make the poster image cognitively salient, participants were first prompted to write a brief description of their surroundings and how it made them feel. This packet, which included the same two measures of dispositional prosociality from Experiment 1 (SRAS: α = 0.63; virtues scale: α = 0.83), was intentionally kept brief (i.e., it was completed by participants in less than 10 min), and participants signed up for a 30-min research time-slot, leaving them 20 additional min to potentially help (and avoid the possibility that they did not help because they did not have time).

Following completion of the packet, participants were told that they had completed the study, but if they would like to help, they could participate in a 20-min pilot study (for no additional credit) that was still “in development.” They also were told that their assistance would be extremely helpful to the researchers. If participants agreed to help, they were directed into another room to complete a boring task of rating up to 60 geometric shapes (e.g., hexagons) along several dimensions (e.g., “how geometrically soothing is the image?”). The experimenter informed them that they could stop at any time. After rating each shape on several dimensions, participants read a screen that thanked them for helping, and asked if they would like to continue helping by rating the next shape. This continued for up to 60 iterations. All participants were stopped after a maximum of 15 min if they were still working on the task. Upon cessation or when they were stopped by the experimenter, participants completed the MLQ measure of meaning in life (α = 0.89). Finally, participants indicated what they thought the nature of the study was about (no participants guessed correctly) and were fully debriefed.

**FIGURE 1:**
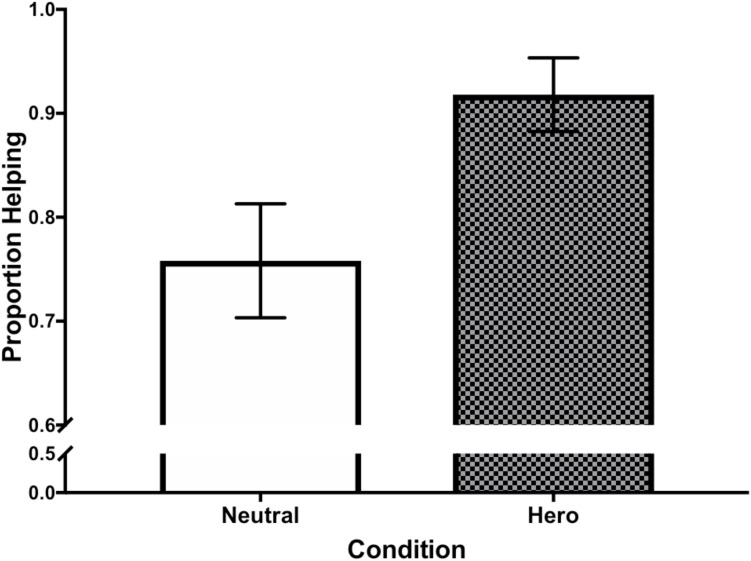
A subtle superhero prime increased the likelihood of helping in Experiment 2.

### Results

We examined whether the priming condition affected actual helping behavior and self-reported meaning in life. Providing support for Hypothesis 1, participants primed with the superhero poster were significantly more likely to help (91.80%) than those primed with the neutral poster (75.80%), χ^2^ = 5.78, *p* = 0.016 (see Figure 1)^1^. Contrary to Hypothesis 2, there was no effect of priming condition on meaning in life, *t*(121) = 0.57, *p* = 0.571, *d* = 0.10. Moreover, there was no effect of helping (vs. not helping) on meaning in life, *t*(121) = 0.22, *p* = 0.832, *d* = 0.04.

As in experiment 1, the effect on condition on helping behavior was not moderated by self-reported virtuousness [*F*(1,119) = 0.03, *p* = 0.877] or self-reported altruism [*F*(1,119) = 0.03, *p* = 0.868]. Thus, once again, the prime appears to work similarly for people of varying levels of dispositional prosociality. The subtle prime did not affect self-reported virtuousness [*F*(1,121) = 1.69, *p* = 0.196] or altruism [*F*(1,227) = 0.047, *p* = 0.829].

### Discussion

The results of Experiment 2 demonstrated that subtle priming of superhero images was associated with increased helping behavior, confirming our hypothesis and conceptually replicating Experiment 1. Those primed with a Superman poster were significantly more likely to help than those primed with a bicycle. Interestingly, the prime influenced the decision to help, but did not influence the amount of helping. Obviously, that initial decision point is the most critical and future research should investigate more closely how heroes and images of heroes might influence different aspects of prosocial behaviors.

However, partially inconsistent with the findings of Experiment 1, the priming images had no significant effects on meaning in life. We see several possible reasons for this. First, the helping behavior may not have been perceived by the participant as particularly valuable; simply assisting an experimenter may not serve the same function as coming the aid of someone in need. Thus, the link between this particular helping behavior and meaning may be rather weak. A second possibility is that there may have been too much time between the prime and the assessment of meaning in life, given that the helping behavior took a relatively long amount of time (e.g., Dijksterhuis and Bargh, 2001). Nonetheless, these results confirmed the hypothesis that even a subtle superhero priming would increase prosocial behavior in participants. The motivational nature of superheroes appears to increase helping behavior.

## General Discussion

Exposure to heroes can confer both intrapersonal and interpersonal benefits. Two experiments examined the variation in the effect of heroes on prosocial intentions and behaviors, as well as meaning in life. Experiment 1 demonstrated that subtle priming of superheroes increases prosocial intentions: after viewing images with superhero symbols embedded into them, participants reported greater likelihood to help in hypothetical situations in which people were in need. These helping intentions were associated with meaning in life. Experiment 2 demonstrated that subtly priming a superhero (i.e., Superman) via an image led to increased behavioral helping. However, contrary to predictions and the results of Experiment 1, the priming of a heroic image did not influence meaning in life.

These experiments highlight how even the subtle activation of heroic constructs through visual images of superheroes may influence intentions to help as well as actual helping behavior. Although we drew attention to the superhero image as a way of activating related constructs in the minds of the participants, such exposure was undoubtedly less potent than other behaviors that involve greater cognitive attention (e.g., watching a movie, reading a comic book)–this makes it a strong test of our hypothesis. These priming effects need not be explicit to exert an influence on motivations and behaviors. However, future work could advance this work by employing a subliminal priming method, though we return to this point in the General Discussion. This work is consistent with prior work linking heroes and prosociality (Kinsella et al., 2015a,b; Franco et al., 2016) as well as the association between prosociality and meaning (Van Tongeren et al., 2016). Moreover, it suggests that heroes may serve an important cultural purpose of motivating coalitional behavioral that strengthens the prosociality of a society.

### Limitations and Suggestions for Future Research

Building on previous research on prosocial behavior, we sought to understand the influence of fictional heroes on helping behavior and meaning in life. However, heroes come in many forms: real versus fictional, known personally versus unknown, paragons of virtue versus flawed characters. The manner in which we are exposed to heroes also varies: relatively subtle and implicit versus explicit, images versus deliberate thought, etc. Future work should explore the effects of more supraliminal (i.e., explicit) priming of superheroes, such as talking about or writing about a hero, as well as a more subtle activation of constructs, such as using a subliminal priming methodology (e.g., see Van Tongeren and Green, 2010 for an example). Future research also should explore the effects of priming real heroic individuals as well as symbolic/fictional superheroes. As articulated in the introduction, it is possible that some real heroes may confer a threat or social comparison contrast effect under some conditions that might not result in increased helping or meaning in life. Relatedly, past research has found that being primed with extreme exemplars on a judgment category (e.g., Hitler or the Pope on hostility/kindness) may result in contrast effects (Herr, 1986; Dijksterhuis et al., 1998). In sum, we advocate for extending this research into related heroes arenas rather than assuming that these findings necessarily apply across various dimensions of heroes.

Experiment 1 measured helping intentions through self-reported hypothetical helping scenarios. Therefore, in Experiment 2 we sought to measure actual helping behavior by asking participants to participate in a pilot study. A limitation of Experiment 2 was that experimenters were not blind to experimental condition. Although the implicit priming of superheroes did increase helping behavior, the helping behavior did not affect the reported meaning in life (as it did in the Experiment 1). This may have been due to the characteristics of the helping behavior: Participants rated their meaning in life after completing a relatively tedious task of rating abstract pictures on numerous dimensions. Thus, though the task was deemed helpful by the experimenters, it may have left the participants feeling bored rather than feeling like they had actually been helpful. Moreover, in Experiment 1, the helping intention questions related were both *social* (i.e., directed toward another person with whom the participant would hypothetically interact) and *necessary* (i.e., assisting someone in need who would otherwise be disadvantaged). Agreeing to assist a professor, who was not present and who could ostensibly find help from other participants for this task and was not in any danger, may not have qualified as a strong helping behavior in Experiment 2. [It is also possible that our theorizing is not correct, but ample previous research linking prosocial actions and increased meaning in life (e.g., Van Tongeren et al., 2016) makes us first look to flawed methodology.] Future helping behavior should involve stronger or more relational helpful behavior (i.e., donating to charity or helping an individual in need) before measuring meaning in life. Perhaps helping behavior that *rescues* or *saves* is more strongly associated with meaning in life. Future work could explore this possibility.

The experiments here also measured only short-term effects on helping behavior. Future work should examine how heroes affect individuals’ behavior long-term. For example, would chronic activation of heroes via reading a biography or frequently using a mug with a hero’s image on it elicit a relatively enduring increase in prosociality? Relatedly, it may be beneficial to also examine how recalling heroic historical figures (e.g., Rosa Parks, Gandhi, Winston Churchill), may have an effect on helping behavior. Also, we do not know much about the exact mechanism by which these priming effects work; future work could determine which schemas were activated by measuring the cognitive accessibility of related words or concepts (e.g., morality, virtue). Finally, these effects were not moderated by dispositional levels of self-rated prosociality, though these assessments came after the prime and not before it in order to avoid inadvertently activating virtue-related schemas. Future work could examine these constructs before the priming induction.

## Conclusion

Heroes loom large as exemplars of morality. They often embody virtues that we wish to express in our lives. Our findings suggest that heroic images–even relatively subtle images of superheroes–may increase one’s intentions to help and actual helping behavior. As superheroes become an increasing large and accessible part of the symbolic cultural narrative, their role in inspiring virtuous and meaningful lives may become more robust. As this occurs, we may, as Mark Twain wrote, continue our fascination with, and perhaps even worship of, heroes.

## Author Contributions

All authors wrote the manuscript. DVT, AC, and JG led the design of the experiments. DVT oversaw the running of the experiments and conducted the data analyses. RaH, ME, EJ, KD, HN, AS, and RuH ran the experiments.

## Conflict of Interest Statement

The authors declare that the research was conducted in the absence of any commercial or financial relationships that could be construed as a potential conflict of interest.

## Footnotes

1 However, examining those that helped, the priming condition did not affect how long participants helped, *t*(101) = 0.1.24, *p* = 0.218, *d* = 0.25, or how many images they rated, *t*(99) = 0.97, *p* = 0.334, *d* = 0.20. Moreover, when including only those participants in the experimental condition who explicitly wrote about the Superman poster, the effect remained significant, χ^2^ = 6.33, *p* = 0.012.
